# Serum amyloid P inhibits granulocyte adhesion

**DOI:** 10.1186/1755-1536-6-2

**Published:** 2013-01-17

**Authors:** Anu S Maharjan, David Roife, Derrick Brazill, Richard H Gomer

**Affiliations:** 1 Department of Biochemistry and Cell Biology, MS-140, Rice University, 6100 S Main Street, 77005-1892, Houston, TX, USA; 2 Department of Biology, MS-3474, Texas A&M University, College Station, 77843-3474, TX, USA; 3 Department of Biology, Hunter College, 695 Park Avenue, 10065, New York, NY, USA

**Keywords:** Serum amyloid P, Neutrophil, Adhesion, Acute respiratory distress syndrome, ARDS, ALI

## Abstract

**Background:**

The extravasation of granulocytes (such as neutrophils) at a site of inflammation is a key aspect of the innate immune system. Signals from the site of inflammation upregulate granulocyte adhesion to the endothelium to initiate extravasation, and also enhance granulocyte adhesion to extracellular matrix proteins to facilitate granulocyte movement through the inflamed tissue. During the resolution of inflammation, other signals inhibit granulocyte adhesion to slow and ultimately stop granulocyte influx into the tissue. In a variety of inflammatory diseases such as acute respiratory distress syndrome, an excess infiltration of granulocytes into a tissue causes undesired collateral damage, and being able to reduce granulocyte adhesion and influx could reduce this damage.

**Results:**

We found that serum amyloid P (SAP), a constitutive protein component of the blood, inhibits granulocyte spreading and granulocyte adhesion to extracellular matrix components. This indicates that in addition to granulocyte adhesion inhibitors that are secreted during the resolution of inflammation, a granulocyte adhesion inhibitor is present at all times in the blood. Although SAP affects adhesion, it does not affect the granulocyte adhesion molecules CD11b, CD62L, CD18, or CD44. SAP also has no effect on the production of hydrogen peroxide by resting or stimulated granulocytes, or *N*-formyl-methionine-leucine-phenylalanine (fMLP)-induced granulocyte migration. In mice treated with intratracheal bleomycin to induce granulocyte accumulation in the lungs, SAP injections reduced the number of granulocytes in the lungs.

**Conclusions:**

We found that SAP, a constitutive component of blood, is a granulocyte adhesion inhibitor. We hypothesize that SAP allows granulocytes to sense whether they are in the blood or in a tissue.

## Background

Infections or injuries to tissues such as the lungs cause the damaged cells to recruit immune cells, including granulocytes and monocytes, to the injury site [1,2]. The transmigration of granulocytes such as neutrophils to the site of injury or infection requires the interaction of neutrophils with endothelial cells and extracellular matrices [1,3,4]. In blood vessels, neutrophils are generally quiescent, but after an injury or infection, neutrophils begin to tether and roll on the blood vessel using the selectin family of adhesion molecules such as CD62L, CD62P, and P-selectin glycoprotein ligand-1 (PSGL-1) [5-7]. These adhesion molecules interact with endothelial cell adhesion molecules such as E-selectin, P-selectin, and PSGL-1 [5-7]. Activated endothelial cells also interact with neutrophil glycoproteins such as CD44 and CD43 through E-selectin to slow neutrophil rolling [5,8]. CD44 interacts with E-selectin and causes the redistribution of PSGL-1 or L-selectin on rolling neutrophils, which then promotes the tethering of neutrophils and slows downthe rolling velocity [5]. The slow neutrophil rolling allows neutrophils to sense signals such as interleukin (IL)-8, tumor necrosis factor (TNF)α, granulocyte macrophage colony-stimulating factor (GM-CSF), or *N*-formyl-methionine-leucine-phenylalanine (fMLP) from damaged cells or infection [5,9-13], and activate integrin adhesion molecules such as CD11b and CD18 [6,7,9,14-16]. IL-8 is a neutrophil chemoattractant that can induce neutrophil degranulation and enhance neutrophil production of reactive oxygen species [9,16]. TNFα and GM-CSF increase neutrophil adherence, release of reactive oxygen species, and phagocytosis [10-13]. fMLP resembles bacterial waste products, and activates neutrophil chemotaxis [17-20].

The upregulation of the adhesion molecules CD11b and CD18 let neutrophils interact with endothelial ligands such as intercellular adhesion molecule 1 (ICAM-1), which causes neutrophils to firmly adhere to the endothelium [7] and move through the blood vessel into an injured site [15]. Integrin molecules such as CD11b and CD18 can also bind to extracellular matrix components such as fibronectin, fibrinogen, laminin, and collagen, and this binding aids in the movement of neutrophils through extracellular matrices [1,3,4]. Other integrin adhesion molecules such as CD61 facilitate leukocyte migration, but little is known about their roles in neutrophil migration. Once activated neutrophils are at injured sites, they can release reactive oxygen species and proteases, and then engulf bacteria and debris by phagocytosis [21,22].

In the normal resolution of wound healing, activated granulocytes such as neutrophils undergo programmed cell death, which prevents the release of reactive oxygen species from the granulocytes, thereby preventing any cell damage in the surrounding tissue [23]. Since activated granulocytes can damage surrounding cells, cytokines such as IL-4 and IL-10 inhibit excessive recruitment of granulocytes into the site of injury [24-27]. IL-4 and IL-10 inhibit the production of IL-8 and the release of TNFα and IL-1β, which in turn limits granulocyte accumulation and activation [24,25,27]. Lipid mediators such as lipoxin A_4_ (LXA_4_) and lipoxin B_4_ (LXB_4_) inhibit neutrophil recruitment by reducing neutrophil adhesion to endothelial cells and vascular permeability [28,29]. Other lipid mediators including D-series and E-series resolvins and protectins also inhibit transendothelial migration of neutrophils [30,31].

Secreted pentraxin proteins such as pentraxin-3 (PTX3) and C-reactive protein (CRP) also limit neutrophil recruitment to a site of injury [32-37]. PTX3 is a pentraxin that is produced and released by monocytes, dendritic cells, endothelial cells, and smooth muscle cells in response to inflammatory signals such as IL-1β, TNFα, or Toll-like receptor (TLR) agonists [38]. CRP is a pentraxin secreted into the blood by the liver as an acute phase protein in humans, and inhibits neutrophil adhesion and chemotaxis on activated endothelial cells [33,36]. Neutrophils recognize the pentraxin family of proteins through Fcγ receptors [39,40]. Neutrophils express high levels of FcγRII (CD32) and FcγRIII (CD16), and express low or undetectable levels of FcγRI (CD64) [41,42]. These receptors bind to the Fc portion of IgG immunoglobulins [43] or pentraxin proteins such as PTX3, CRP, and serum amyloid P (SAP), and help in the opsonization and phagocytosis of bacteria or debris [44-47]. Serum amyloid P is a pentraxin that is constitutively secreted into the blood by the liver [44]. The circulating SAP levels are approximately 30 μg/ml in humans [48], and approximately 15 μg/ml in C57BL/6 mice [49]. SAP effectively inhibits the differentiation of monocytes to fibrocytes [50,51] through FcγRI and FcRγ [51]. *In vivo*, injections of SAP significantly reduce bleomycin-induced pulmonary fibrosis in mice and rats [52]. Although it has been reported that SAP elicits antifibrotic activity by stimulating IL-10 [53], a recent study has shown that highly purified SAP does not stimulate IL-10 production [54].

Little is known about SAP’s interaction with granulocytes, which have Fcγ receptors. Activated granulocytes release reactive oxygen species such as hydrogen peroxide and superoxide anions through activation of NADPH oxidase [55] to kill microbes such as bacteria. However, excessive release of these cytotoxic products can further damage an injured tissue. SAP appears to decrease neutrophil oxygen metabolism [56], but SAP has no effect on the production of hydrogen peroxide by neutrophils stimulated by digitonin, mistletoe lectin, or fMLP [57]. IL-8 is a chemoattractant, and SAP has been reported to bind IL-8 [58]. In the presence of IL-8, SAP decreases neutrophil binding to fibronectin coated plates [58]. However, in the absence of IL-8, SAP acts as a neutrophil chemoattractant and increases neutrophil adhesion [58]. SAP increases the percentage of neutrophils expressing adhesion molecules such as CD11b and CD18 and the fibronectin receptor α5β1 [58]. In flow chambers, SAP inhibits the binding of human neutrophils to TNFα-stimulated human umbilical vein endothelial cells [59]. Since granulocytes can recognize SAP through Fc receptors, and the reports of the regulation of neutrophil adhesion by SAP seem inconsistent, we examined the effect of SAP on granulocyte adhesion and recruitment to sites of inflammation.

## Materials and methods

### Isolating peripheral blood mononuclear cells (PBMCs) or granulocytes

Blood was collected from healthy adult volunteers with specific approval from the Institutional Review Boards of Rice University and Texas A&M University. Written consent was received and all samples were deidentified before analysis. PBMCs were isolated and incubated in RPMI serum-free medium (SFM) as described previously [60]. Granulocytes were isolated from blood using Lympholyte-poly (Cedarlane Laboratories, Hornby, Canada) following the manufacturer’s directions and resuspended in RPMI-1640 (Sigma) or 2% bovine serum albumin (BSA) (Fraction V, A3059, Sigma) in RPMI-1640. To check the purity of the granulocytes, 100 μl of the isolated granulocytes were analyzed by flow cytometry (Accuri Cytometers, Ann Arbor, MI, USA) using the combination of forward scatter (correlates to cell size) and side scatter (correlates to cell granularity). Isolated granulocytes were larger and more granular than other cells. As an additional check of granulocyte purity, 200 μl of 0.5 × 10^6^ cells/ml granulocytes in 2% BSA-RPMI was aliquoted into a well of an eight-well glass chamber slides (Lab-Tek, Nalge Nunc International, Naperville, IL, USA) for 1 h at 37°C. After incubation, 150 μl of media was removed and the slide was spun at 400 *g* for 5 minutes using a cytospin centrifuge (Shandon, Runcorn, UK). The cells were then fixed with 200 μl of 2% paraformaldehyde (PFA) in phosphate-buffered saline (PBS) for 15 minutes at room temperature. After the PFA was removed, 400 μl of ice-cold methanol was added to the wells for 1 h at 4°C to permeabilize the cells. After gently removing the methanol, 400 μl of PBS was added to the wells for 10 minutes at room temperature and then gently pipetted out from the corner of the well. This was repeated twice. The slide was then mounted with a 4',6-diamidino-2-phenylindole (DAPI)-containing mounting media (Vectashield, Vector Laboratories). Images of the cells were captured on an Axioplan2 microscope (Zeiss) with a CoolSNAP HQ digital camera (Photometrics, Tucson, AZ, USA) and Metamorph software (Molecular Devices, Dowington, PA, USA).

### Production of human SAP or murine SAP

Human SAP (hSAP) was from Calbiochem (Calbiochem-EMD Chemicals, Darmstadt, Germany). Commercial human SAP was buffer exchanged with 20 mM sodium phosphate buffer as described previously [60]. Human SAP or murine SAP (mSAP) were also prepared from commercially available human serum (Gemini, West Sacramento, CA, USA) or murine serum (Gemini) using calcium-dependent binding to phosphoethanolamine-conjugated agarose as described previously [52]. Commercial or purified SAP was stored at 1 mg/ml in 20 mM sodium phosphate buffer, pH 7.4 at −20°C.

### Granulocyte spreading assay with cell debris

PBMCs at 1 × 10^6^ cells/ml in SFM were lysed with a Dounce homogenizer and a drill-driven Teflon pestle (Thomas Scientific, Swedesboro, NJ, USA) at 300 RPM for 60 strokes to make cell debris. Then, 100 μl of PBMCs at 0.5 × 10^6^ cells/ml were incubated in flat bottom 96-well tissue culture plates (BD, Franklin Lakes, NJ, USA) in the presence or absence of 100 μl of undiluted debris at 37°C. After 7 days, the supernatants were clarified by centrifugation at 10,000 *g* for 10 minutes. Supernatants were collected into Eppendorf tubes and flash frozen with liquid nitrogen, and stored at −80°C until further use. A total of 100 μl of 5 × 10^5^ cells/ml granulocytes were incubated in 20 μg/ml SAP in RPMI, 25% PBMC supernatant in RPMI, a mix of 25% PBMC supernatant and 20 μg/ml SAP in RPMI, or in RPMI. After 1 h, fields of granulocytes were photographed using a phase-contrast microscope with a 20 × objective. Granulocytes and spread granulocytes were then counted.

### Granulocyte adhesion

Wells of flat bottom 96-well tissue culture plates (BD) were precoated with 50 μl of 20 μg/ml bovine plasma fibronectin (Sigma) in PBS or 20 μg/ml cellular human foreskin fibroblast fibronectin (Sigma) in PBS for 1 h at 37°C. After removing the fibronectin, the wells were washed three times with 200 μl of PBS and then blocked with 200 μl of 2% BSA-PBS for 2 h at room temperature. The wells were then washed three times with 200 μl of PBS and once with 200 μl of 2% BSA-RPMI before adding granulocytes. A total of 500 μl of granulocytes at 1 × 10^6^ cells/ml in 2% BSA-RPMI were incubated in an Eppendorf tube (preincubated with 2% BSA-RPMI for 2 h at 37°C), and SAP (or an equal volume of buffer) was added to a final concentration of 30 μg/ml for 30 minutes at 37°C. A total of 100 μl of 1 × 10^6^ cells/ml granulocytes was then incubated in the well of a 96-well plate for 10 minutes at 37°C to allow granulocytes to settle. Then, 1 μl of 10 μg/ml recombinant human TNFα (Peprotech, NJ, USA) in 2% BSA-RPMI was then added to the well and gently mixed by stirring with the pipette tip. After a 30-minute incubation with TNFα at 37°C, non-adherent granulocytes were removed and the wells were washed three times by pipetting in and then removing 100 μl of 37°C PBS. The plate was then air dried, stained with methylene blue and eosin (Richard-Allan Scientific, Kalamazoo, MI, USA) [61], and the number of adherent granulocytes was counted in five different 900 μm diameter fields of view. For assays on dry fibronectin, the granulocytes adhesion was carried out as above except the plates were air dried after blocking with BSA.

### Staining for granulocyte adhesion molecules

A total of 500 μl of granulocytes at 2.0 × 10^6^ cells/ml were aliquoted into Eppendorf tubes (precoated with 2% BSA-RPMI for 1 h at 37°C) and incubated with 10 ng/ml or 1 ng/ml TNFα, 100 ng/ml IL-8, or 10 ng/ml or 1 ng/ml GM-CSF in the presence or absence of 10 μg/ml or 60 μg/ml SAP for 1 h at 37°C. For the granulocytes that were stained with (anti-human) anti-CD18, anti-CD61, or anti-CD44, SAP was added to 30 μg/ml. Cells were then washed with ice-cold PBS, collected by centrifugation at 500 *g* for 5 minutes, and resuspended in 1 ml of 4% BSA-PBS. Cells were stained in BSA-coated tubes with 5 μg/ml antibodies against CD11b (BioLegend, San Diego, CA, USA), CD62L (BD Biosciences), CD32 (BD Biosciences), CD18 (BioLegend), CD61 (BD Biosciences), CD44 (BD Biosciences), or mouse IgG1 isotype control (BioLegend) for 30 minutes at 4°C. The cells were then washed three times in ice-cold PBS, and incubated with 2.5 μg/ml fluorescein isothiocyanate (FITC)-conjugated F(ab′)2 goat anti-mouse IgG antibodies (crossadsorbed against human Ig, Southern Biotechnology, Birmingham, AL, USA) as described previously [60,62]. The cells were washed three times in ice-cold PBS, resuspended in 200 μl 4% BSA-PBS, and analyzed by flow cytometry.

### Hydrogen peroxide production

Wells of black 96-well cell culture plates (Nalge Nunc, Rochester, NY, USA) were precoated with 50 μl of 20 μg/ml plasma fibronectin for 1 h at 37°C. The fibronectin was then removed, and the wells were washed three times with 200 μl of PBS, and then washed once with Krebs-Ringer phosphate glucose buffer (KRPG) (145 mM NaCl, 4.9 mM KCl, 0.54 mM CaCl_2_, 1.2 mM MgSO_4_, 5.8 mM sodium phosphate, and 5.5 mM glucose, pH 7.35) [11]. A total of 500 μl of granulocytes at 1.5 × 10^6^ cells/ml in KRPG were incubated in an Eppendorf tube (preincubated with 2% BSA-KRPG for 2 h at 37°C) and SAP was added to a final concentration of 30 μg/ml. As a control, a similar tube had an equal volume of buffer added to it. These were incubated for 30 minutes at 37°C. An assay mixture of 100 μl of KRPG, 20 μl of 300 μM scopoletin (Sigma) in KRPG, 20 μl of 10 mM NaN_3_ in KRPG, and 20 μl of 10 U/ml horseradish peroxidase (Sigma) in KRPG were aliquoted into a well and the plate was equilibrated to 37°C for 5 minutes as described previously [11,63]. Then, 20 μl of granulocytes incubated with or without 30 μg/ml SAP was added to the assay mixture in the presence or absence of 20 μl of 1 μg/ml TNFα in KRPG, 20 μl of 1 μM fMLP (Sigma) in KRPG, 20 μl of 1 μM phorbol 12-myristate 13-acetate (PMA) (Sigma) in KRPG, 20 μl of 1 μM phorbol 12,13-dibutyrate (PDBu) (Sigma) in KRPG, or 20 μl of KRPG. The 96-well plate was incubated at 37°C and the fluorescence (excitation: 360 nm emission: 460 nm) was monitored every 10 minutes for 3 h using a Synergy MX plate reader (BioTek, Winooski, VT, USA).

### Transmigration of granulocytes

A total of 50 μl of granulocytes at 1 × 10^6^ cells/ml in 2% BSA-RPMI was added to the top chamber of a 3 μm pore size nylon membrane insert in a 24 well plate (BD) in the presence or absence of 10 nM fMLP, 30 μg/ml SAP, 10 nM fMLP and 30 μg/ml SAP or an equal volume of buffer in 2% BSA-RPMI. The bottom chambers contained 600 μl of 10 nM fMLP in 2% BSA-RPMI, 600 μl of 30 μg/ml SAP in 2% BSA-RPMI, 600 μl of 10 nM fMLP and 30 μg/ml SAP in 2% BSA-RPMI, or equal volumes of buffer in 2% BSA-RPMI. The transmigration was carried out for 2 h at 37°C. The top chamber was removed, and the granulocytes that had migrated into the bottom chamber were then counted with a flow cytometer.

### Staining for apoptotic granulocytes

A total of 500 μl of granulocytes at 2.0 × 10^6^ cells/ml were aliquoted into Eppendorf tubes (precoated with 2% BSA-RPMI for 1 h at 37°C) and incubated with 10 ng/ml or 1 ng/ml TNFα, or 10 ng/ml or 1 ng/ml GM-CSF in the presence or absence of 60 μg/ml SAP for 22 h at 37°C. The cells were then washed with ice-cold PBS, collected by centrifugation at 500 *g* for 5 minutes, and resuspended in 1 ml of 4% BSA-PBS. Cells were stained with 5 μg/ml Alexafluor 488-conjugated annexin V (Invitrogen) for 30 minutes at 4°C. The cells were then washed three times in ice-cold PBS, resuspended in 200 μl 4% BSA-PBS, and analyzed with a flow cytometer.

### Murine granulocyte adhesion assay

C57/BL6 mice (4 weeks old; Jackson Laboratories, Bar Harbor, ME, USA) were housed at the Laboratory Animal Resources and Research facility at Texas A&M University. Animal procedures were approved by the Institutional Animal Care and Use Committee at Texas A&M University. Mice were killed and blood was obtained via cardiac puncture. From two to three mice, a total of 2 to 3 ml of blood was collected in an ethylenediaminetetra-acetic acid (EDTA)-containing vacutainer tube (BD) and the red blood cells (RBC) in 2 ml of blood were lysed by adding 1 ml of ammonium chloride/potassium bicarbonate (ACK) lysis buffer (15 mM NH_4_Cl, 1 mM KHCO_3_, 0.01 mM Na_2_EDTA) and incubating for 3 minutes at room temperature. Cells were collected by centrifugation at 500 *g* for 5 minutes at room temperature. The pellets were resuspended in 200 μl PBS, and 1 ml ACK lysis buffer was added. After 3 minutes, cells were collected by centrifugation. This was then repeated two additional times. Cells were resuspended in 1 ml PBS and then collected by centrifugation. The cells were then resuspended in 1 ml of 2% BSA-RPMI. Wells of flat bottom 96-well tissue culture plates (BD) were precoated with 50 μl of 20 μg/ml plasma fibronectin (Sigma) in PBS for 1 h at 37°C. A granulocyte adhesion assay was carried out in 2% BSA-RPMI similar to the human granulocyte adhesion assay using 60 μg/ml human SAP instead of 30 μg/ml. The adhered cells were stained for Ly6G to distinguish granulocytes from other cell types as described previously [64]. The number of adhered Ly6G-positive granulocytes was then counted as described above.

### Granulocyte influx in mice

C57/BL6 mice (4 weeks old; Jackson) were treated with an oropharyngeal aspiration of 50 μl of 0.2 U/kg or 3 U/kg bleomycin (Calbiochem) [65]. The successful aspiration of bleomycin into the lungs was confirmed by listening to the crackling noise heard after the aspiration. At 24 and 48 h following bleomycin aspiration (days 1 and 2), mice were given an intraperitoneal injection of 50 μl of 1 mg/ml hSAP or 1 mg/ml mSAP in 20 mM sodium phosphate buffer or an equal volume of 20 mM sodium phosphate buffer. Mice were killed at day 3 after bleomycin aspiration, and the lungs were perfused with 400 μl of PBS three times to collect cells by bronchoalveolar lavage (BAL) as described previously [66]. The cells were collected by centrifugation at 500 *g* for 5 minutes, and the supernatants were transferred to Eppendorf tubes. The pooled supernatants were flash frozen with liquid nitrogen, and stored at −80°C until further use. The cells collected from BAL were resuspended in 100 μl of 4% BSA-PBS and counted with a hemacytometer. The cells were then diluted in a total volume of 600 μl of 4% BSA-PBS. Then, 100 μl of diluted cells were aliquoted into cytospin funnels and were spun onto glass slides (Superfrost plus white slides, VWR, West Chester, PA, USA) at 400 *g* for 5 minutes using a cytospin centrifuge (Shandon, Cheshire, UK). These cells were then air dried, and stained with 5 μg/ml anti-mouse Ly6G (BioLegend) as previously described [64]. After staining the cells, the number of cells positive for Ly6G per 200 cells was counted. The percentage of positive cells was then multiplied by the total number of cells recovered from the BAL to obtain the number of granulocytes in the BAL. The mice were used in accordance with guidelines published by the National Institutes of Health, and the protocol was approved by the Texas A&M University Animal Use and Care Committee.

### Immunohistochemistry

After BAL, lungs were inflated with prewarmed optimal cutting temperature (OCT) compound (VWR) and then embedded in OCT, frozen on dry ice, and stored at −80°C as described previously [52]. Lung tissue sections (6 μm) were prepared and immunohistochemistry was performed as described previously [52] except slides were incubated with 2.5 μg/ml primary antibodies in 4% BSA-PBS for 60 minutes. The lung sections were stained for Ly6G (BioLegend) to detect granulocytes, CD11b (BioLegend) to detect macrophages, and CD45 (BioLegend) to detect all leukocytes. Isotype-matched mouse irrelevant antibodies were used as controls. Slides were then washed three times with PBS over 30 minutes and incubated with 1.25 μg/ml biotinylated mouse F(ab’)_2_ anti-rat IgG in 4% BSA-PBS for 30 minutes. Slides were then washed three times in PBS over 30 minutes and incubated with a 1:500 dilution of streptavidin alkaline phosphatase (Vector Laboratories) in 4% BSA-PBS for 30 minutes. Staining was developed with a VectorRed Alkaline Phosphatase Kit (Vector Laboratories) for 10 minutes. Slides were then mounted as described previously [52].

### Statistics

Statistical analysis was performed using Prism (GraphPad Software, San Diego, CA, USA). Statistical significance was determined using either analysis of variance (ANOVA) or t test, and significance was defined as *P* < 0.05.

## Results

### Characterization of isolated granulocytes

**Figure 1:**
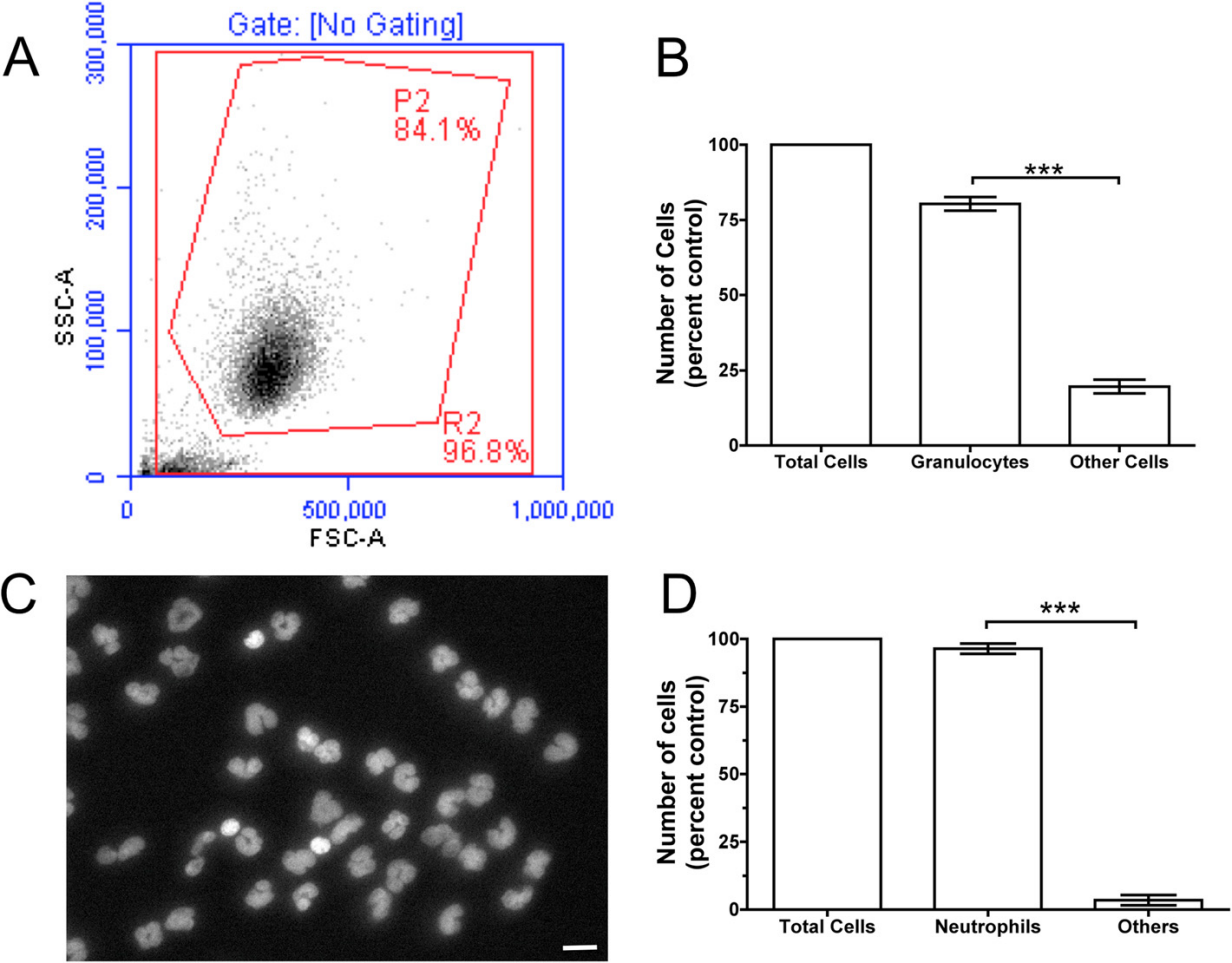
**Characterization of isolated granulocytes.** (**A**) Flow cytometry showing forward-scatter (x-axis) and side-scatter characteristics (y-axis) from 10,000 events. The P2 area shows the granulocyte population. (**B**) The number of granulocytes isolated exceeds the number of other non-granulocyte cells. The results are mean ± SEM of percentage total cells (n = 3 separate experiments). (**C**) Human granulocytes were seeded on a glass slide and spread by centrifugation. Cells were stained with 4',6-diamidino-2-phenylindole (DAPI) to visualize nuclei. Bar is 20 μm. (**D**) The number of granulocytes was counted based on nuclear morphology. There were fewer non-granulocyte cells compared to the isolated granulocytes. The results are mean ± SEM of percentage total cells (n = 3 separate experiments). ***P <0.001 (t test).

Human granulocytes were isolated with lympholyte poly. This isolation technique generated 80 ± 2% pure granulocytes, with small particulate matter accounting for much of the contamination (Figure 1A). The number of non-granulocyte cells was significantly lower than the number of granulocytes obtained (Figure 1B). When isolated cells were further analyzed for their nuclear morphology using DAPI staining, the number of granulocytes was significantly more than the number of non-granulocyte cells (Figure 1C,D). Eosin/methylene blue staining of the isolated granulocytes indicated that there were consistently less than 4% eosinophils and no detectable basophils, with the majority of the cells neutrophils (D Pilling, Texas A&M University, College Station, Texas USA, personal communication). These results suggest that lympholyte poly effectively isolates granulocytes from whole blood.

### SAP inhibits granulocyte spreading

**Figure 2:**
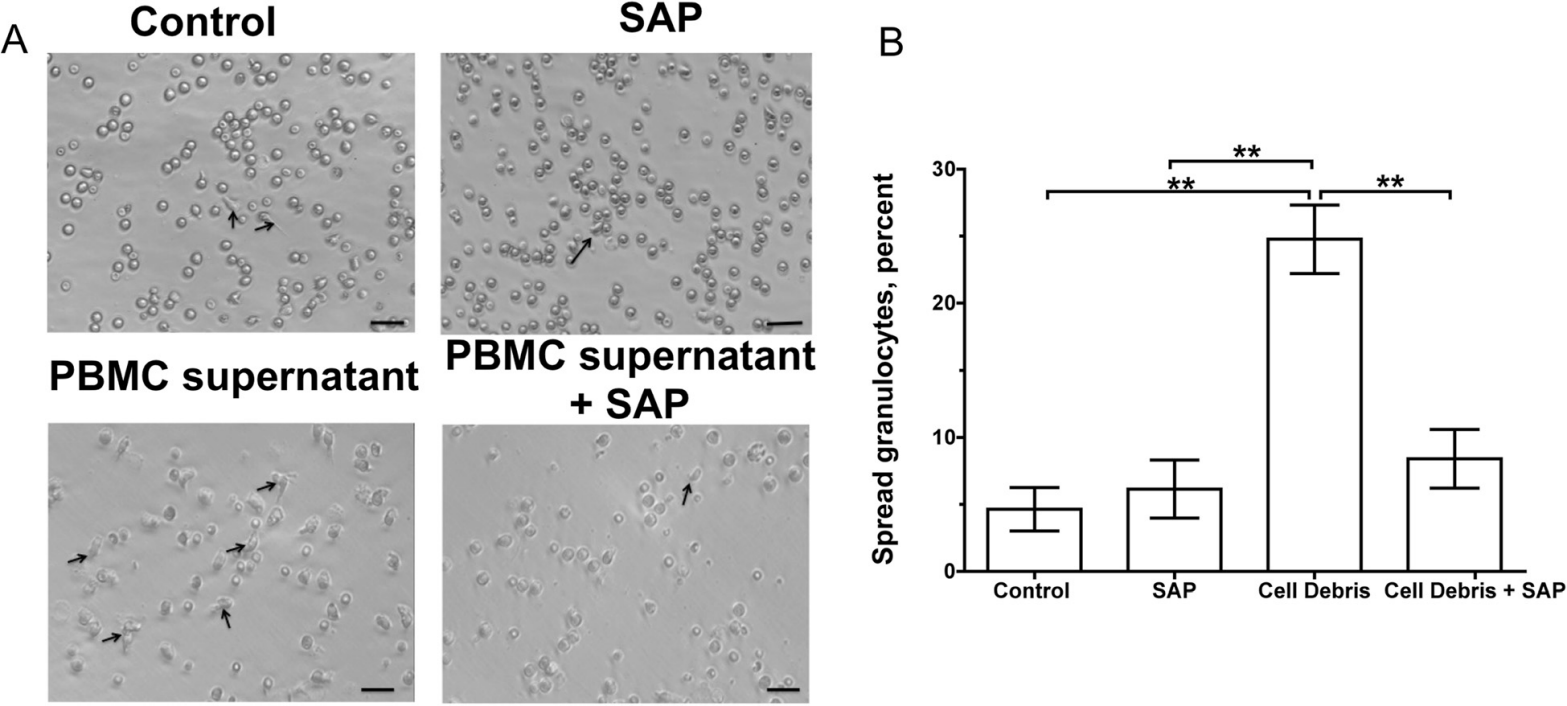
**Serum amyloid P (SAP) inhibits granulocyte spreading.** Human peripheral blood mononuclear cells (PBMCs) were isolated and incubated in the presence of cell debris. After 7 days, the PBMC supernatant was removed. Human granulocytes were incubated in the presence or absence of SAP, PBMC supernatant (cell debris), or the combination of PBMC supernatant and SAP for 1 h. (**A**) Fields of granulocytes were then photographed. Arrows indicate spread granulocytes. All of the figures represent one of three separate experiments. Bar is 20 μm. (**B**) The percentage of the granulocytes that were spread was then counted. Values are mean ± SEM, n = 3. **P <0.01 (one-way analysis of variance (ANOVA), Tukey’s test).

Granulocyte spreading allows granulocytes to polarize and migrate towards the site of injury [67]. To determine the effect of SAP on granulocyte spreading, human PBMCs were incubated with cell debris. The PBMC supernatant (which should contain cell debris as well as PBMC-derived signals elicited in response to debris) was added to human granulocytes in the presence or absence of SAP. We observed spreading granulocytes in the controls, which could have been an effect of laying granulocytes directly on a plastic tissue culture plate (Figure 2A). SAP appeared to decrease granulocyte spreading (Figure 2A). PBMC supernatant increased the numbers of spreading granulocytes (Figure 2A,B), and the addition of SAP inhibited the PBMC supernatant-induced granulocyte spreading (Figure 2B). These results suggest that SAP inhibits granulocyte spreading.

### SAP inhibits human granulocyte adhesion

**Figure 3:**
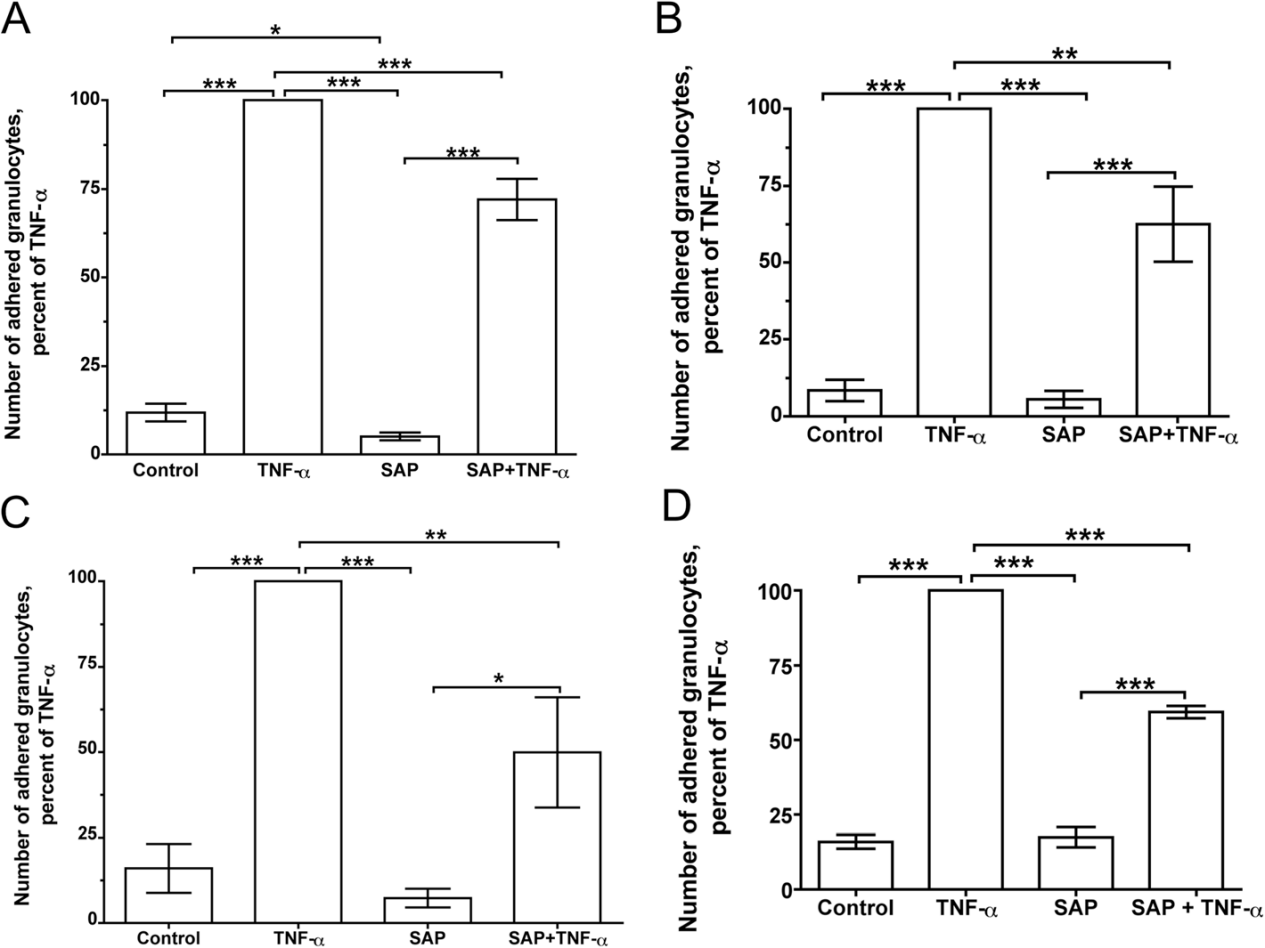
**Serum amyloid P (SAP) inhibits granulocyte adhesion.** (**A**) First, 96-well tissue culture plates were coated with plasma fibronectin. Human granulocytes were treated with or without SAP and/or human tumor necrosis factor (TNF)α, incubated in the plate, and adhered granulocytes were counted. Values are mean ± SEM, n = 23. (**B**) A similar assay was performed using plates coated with cellular fibronectin. Values are mean ± SEM, n = 5. (**C**) A similar assay was performed using plates coated with bovine serum albumin (BSA). Values are mean ± SEM, n = 4. (**D**) A similar assay was performed using plates coated with cellular fibronectin, and then air dried. Values are mean ± SEM, n = 3. *P <0.05, **P <0.01, ***P <0.001 (one-way analysis of variance (ANOVA), Tukey’s test).

TNFα increases the adherence of granulocytes on a variety of extracellular matrices [11,68]. Since SAP inhibited granulocyte spreading, we examined the effect of SAP on granulocyte adhesion. Human granulocytes were incubated with or without SAP, and were then incubated with or without TNFα in plates precoated with plasma fibronectin, cellular fibronectin, BSA, or air-dried cellular fibronectin. As observed previously, incubation with TNFα increased the number of granulocytes that adhered to the wells (Figure 3A-D). In the absence of TNFα, SAP decreased the number of adhered granulocytes to 43.0 ± 7.4% compared to the control (mean ± SEM, n = 23, *P* <0.001 by t test) on plasma fibronectin and decreased the number of adhered granulocytes to 64.1 ± 14.9 compared to the control on cellular fibronectin (n = 5, *P* <0.05 by t test). However, in wells coated with BSA or air-dried fibronectin, there was no significant difference between control and SAP treatment. SAP significantly inhibited the TNFα-induced adhesion of granulocytes to plasma fibronectin, cellular fibronectin, BSA, and air-dried cellular fibronectin (Figure 3A-D). These results suggest SAP inhibits granulocyte adhesion on native cellular and plasma fibronectin, and that SAP inhibits TNFα-induced granulocyte adhesion on a wider variety of surfaces.

### SAP inhibits murine granulocyte adhesion

**Figure 4:**
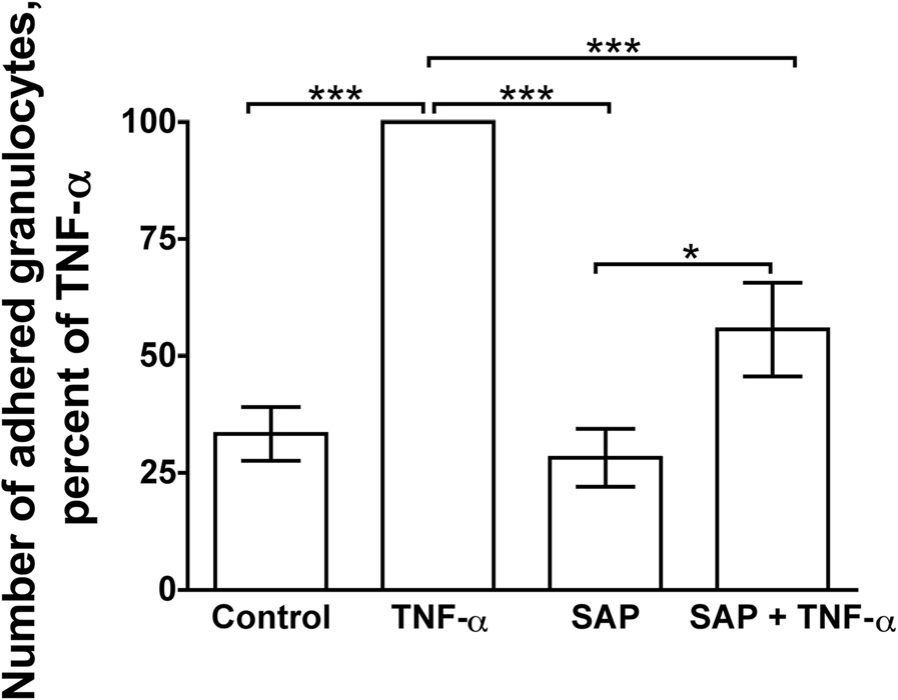
**Serum amyloid P** (**SAP**) **inhibits murine granulocyte adhesion induced by tumor necrosis factor** (**TNF**)**α.** First, 96-well tissue culture plates were coated with plasma fibronectin. Crude murine granulocytes were incubated with SAP and/or TNFα in the plates, and adhered cells were stained for the granulocyte marker Ly6G and counted. Values are mean ± SEM, n = 8. *P <0.05, ***P <0.001 (one-way analysis of variance (ANOVA), Tukey’s test).

Since we found that human SAP can inhibit human granulocyte adhesion induced by TNFα, we examined if human SAP could also inhibit murine granulocyte adhesion. Murine granulocytes were incubated with or without human SAP and TNFα in plates precoated with plasma fibronectin. TNFα increased the number of adhered granulocytes (Figure 4). In the absence of TNFα, SAP had no significant effect on granulocyte adhesion (Figure 4). However, SAP significantly inhibited TNFα-induced granulocyte adhesion to plasma fibronectin (Figure 4).

### SAP has no effect on levels of some adhesion molecules

**Figure 5:**
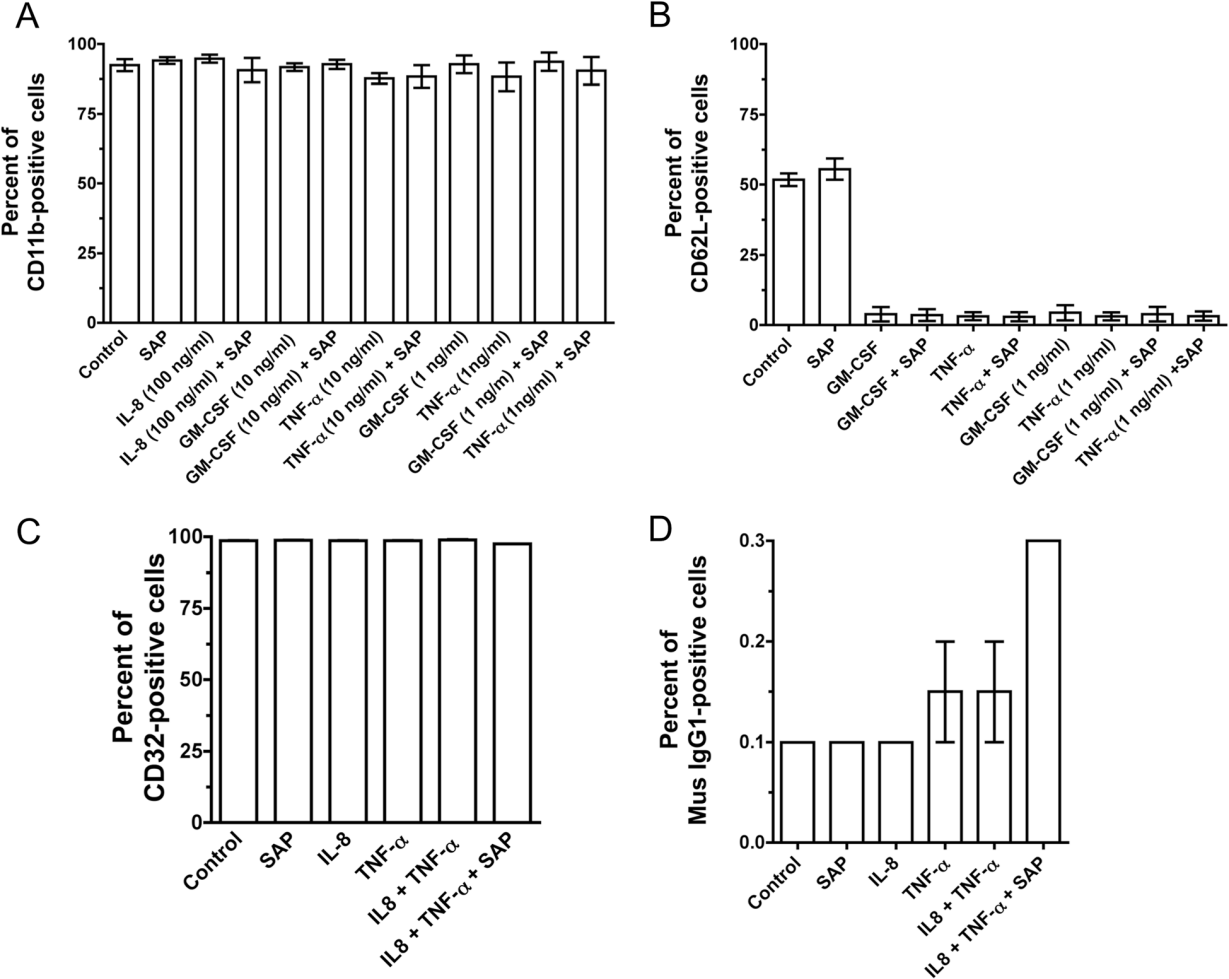
**Serum amyloid P (SAP) has no significant effect on the number of some surface receptors associated with granulocyte activation.** (**A**) Human granulocytes were treated with interleukin (IL)-8, granulocyte macrophage colony-stimulating factor (GM-CSF), or tumor necrosis factor (TNF)α in the presence or absence of SAP. The cells were then stained for CD11b. Values are mean ± SEM, n = 3. (**B**) Granulocytes were treated with GM-CSF or TNFα in the presence or absence of SAP. The cells were then stained for CD62L. Values are mean ± SEM, n = 3. (**C**) Granulocytes were treated with IL-8 or TNFα in the presence or absence of SAP. The cells were then stained for CD32. (**D**) Granulocytes were treated as in (**C**), and were then stained with mouse IgG1. The absence of an error bar indicates that the error was smaller than the line thickness.

**Figure 6:**
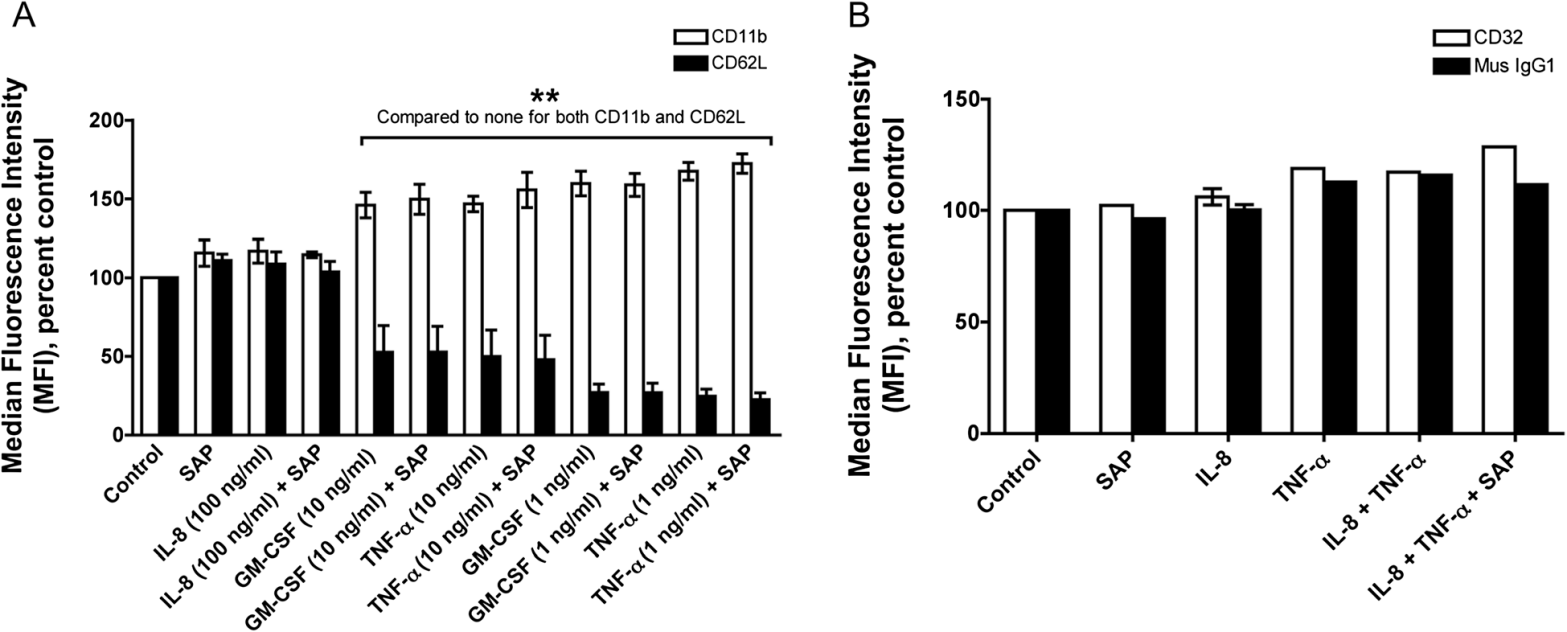
**Serum amyloid P (SAP) has no significant effect on the levels of CD11b, CD62L, or CD32.** (**A**) Human granulocytes were treated as in Figure 4A. The cells were then stained for CD11b or CD62L. Values are mean ± SEM of percentage of median fluorescence intensity of the untreated (none) CD11b-positive or CD62L-positive cells (n = 3 separate experiments except n = 2 for interleukin (IL)-8 and IL-8 + SAP). (**B**) Granulocytes were treated as in Figure 4C, and were then stained for CD32 or stained with a control mouse IgG1. Values are mean ± SEM of percentage of median fluorescence intensity of the untreated (none) CD32-positive or mouse IgG1-positive cells.

To determine how SAP affects adhesion, we analyzed receptors such as CD11b and CD62L whose levels change in activated granulocytes such as neutrophils. Granulocytes were treated with TNFα, IL-8, or GM-CSF in the presence or absence of SAP, and the cells were stained for CD11b, CD62L, and CD32 (FcγRII) as a control. There was no significant effect of any treatment on the number of CD11b-positive cells (Figure 5A). IL-8 in the presence or absence of SAP had no effect on the levels of CD11b or CD62L compared to untreated granulocytes (Figure 6A). As shown previously, TNFα and GM-CSF induced increased levels of CD11b (Figure 6A) [10,13,69], but SAP had no effect on the activation. As observed previously, TNFα and GM-CSF decreased the number and the levels of CD62L-positive cells, [70] but SAP had no effect on the basal or stimulated CD62L levels (Figures 5B and 6A). There was no significant effect on the levels of CD32 or mouse IgG1 (control) staining when granulocytes were treated with TNFα or IL-8 in the presence or absence of SAP (Figures 5C, D and 6B). Together, the data indicate that although TNFα and GM-CSF alter levels of CD11b and CD62L on granulocytes [10,13,69,70], the addition of SAP has no obvious effect on the levels of these adhesion molecules or CD32.

**Figure 7:**
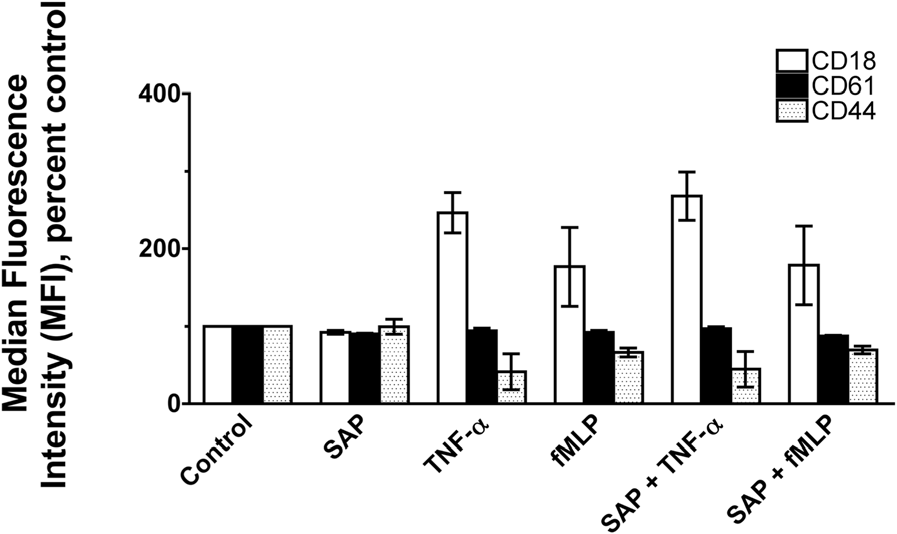
**Serum amyloid P (SAP) has no significant effect on the levels of CD18, CD61, or CD44.** Granulocytes pretreated with or without SAP were incubated in the presence or absence of tumor necrosis factor (TNF)α or *N*-formyl-methionine-leucine-phenylalanine (fMLP). The cells were then stained for CD18, CD61, or CD44. Values are mean ± SEM of median fluorescence intensity of positively stained control cells (n = 3 for CD18 and CD61 and n = 2 for CD44).

Since SAP had no effect on the levels of the surface receptors CD11b or CD62L, we examined the levels of other adhesion molecules such as CD18, CD61, or CD44 [71]. As described previously, TNFα increased the levels of CD18 and decreased levels of CD44 (Figure 7) [10,72], but there was no significant effect of TNFα on the levels of CD61. Similarly, fMLP slightly increased the levels of CD18 (Figure 7) but there was no significant effect on the levels of CD61 or CD44. SAP had no effect on the basal or stimulated levels of CD18, CD44, or CD61. Together, the data indicate that although SAP affects granulocyte adhesion, it does not affect cell surface levels of CD11b, CD62L, CD18, CD61, or CD44.

### SAP has no detectable effect on hydrogen peroxide production

**Figure 8:**
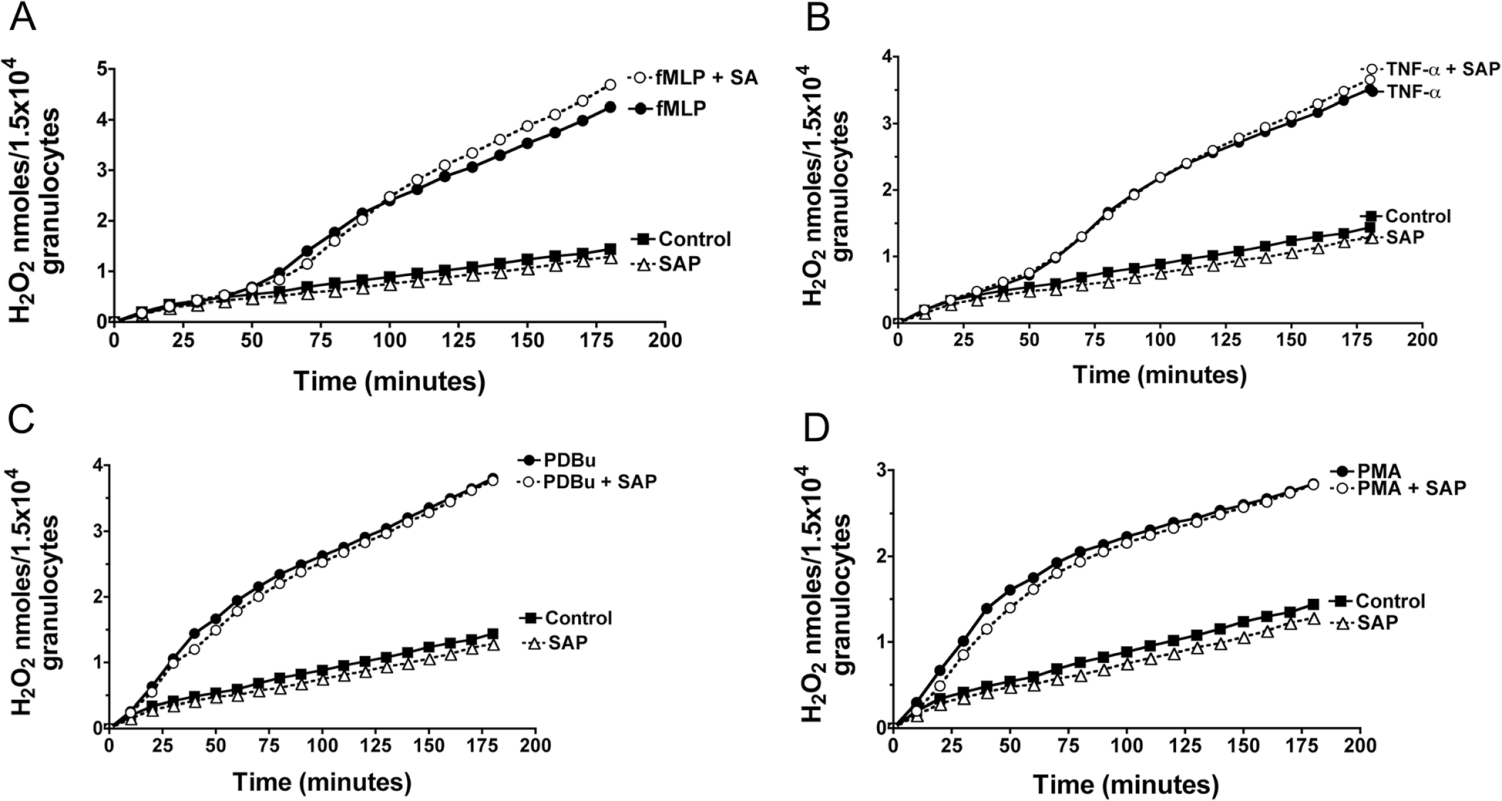
**Serum amyloid P (SAP) has no significant effect on the production of hydrogen peroxide induced by *N-*formyl-methionine-leucine-phenylalanine (fMLP), tumor necrosis factor (TNF)α, phorbol 12**-**myristate 13**-**acetate (PMA), or phorbol 12,13**-**dibutyrate (PDBu).** (**A**) Granulocytes pretreated with or without SAP were treated with fMLP in the presence of scopoletin. The fluorescence intensity of scopoletin was measured over 3 h, and after measuring the effect of different amounts of hydrogen peroxide on scopoletin fluorescence; this was then converted to hydrogen peroxide production. Similar assays were performed using granulocytes stimulated with (**B**) TNFα, (**C**) PDBu, or (**D**) PMA. All of the figures represent one of the three separate experiments for TNFα, fMLP, or PMA, or one of the two separate experiments for PDBu.

Activated granulocytes release hydrogen peroxide to kill microbes such as bacteria [55], but excessive release of hydrogen peroxide can further damage an injured tissue. To examine whether SAP can inhibit the production of hydrogen peroxide from unstimulated granulocytes or granulocytes stimulated by TNFα, fMLP, PDBu, or PMA, we examined the change in the fluorescence intensity of scopoletin, a fluorescent molecule that gets modified by hydrogen peroxide. To inhibit hydrogen peroxide consumption by the granulocytes, this assay uses sodium azide to inactivate myeloperoxidase and catalase, and release the intracellular hydrogen peroxide to the media [73]. As previously observed, in control cells the extracellular hydrogen peroxide levels increased with time, which could have been the release of intracellular hydrogen peroxide since sodium azide will result in the leakage and release of intracellular hydrogen peroxide [73]. Nevertheless, the production of hydrogen peroxide when cells were treated with TNFα, fMLP, PDBu, or PMA exceeded the production of hydrogen peroxide in control cells (Figure 8). As previously observed, SAP had no significant effect on the production of hydrogen peroxide induced by fMLP (Figure 8A). In addition, SAP had no significant effect on the production of hydrogen peroxide induced by TNFα (Figure 8B), PDBu (Figure 8C), or PMA (Figure 8D). Together, the data indicate that although SAP affects granulocyte adhesion, it does not appear to affect granulocyte hydrogen peroxide production.

### SAP has no detectable effect on the migration of granulocytes

**Figure 9:**
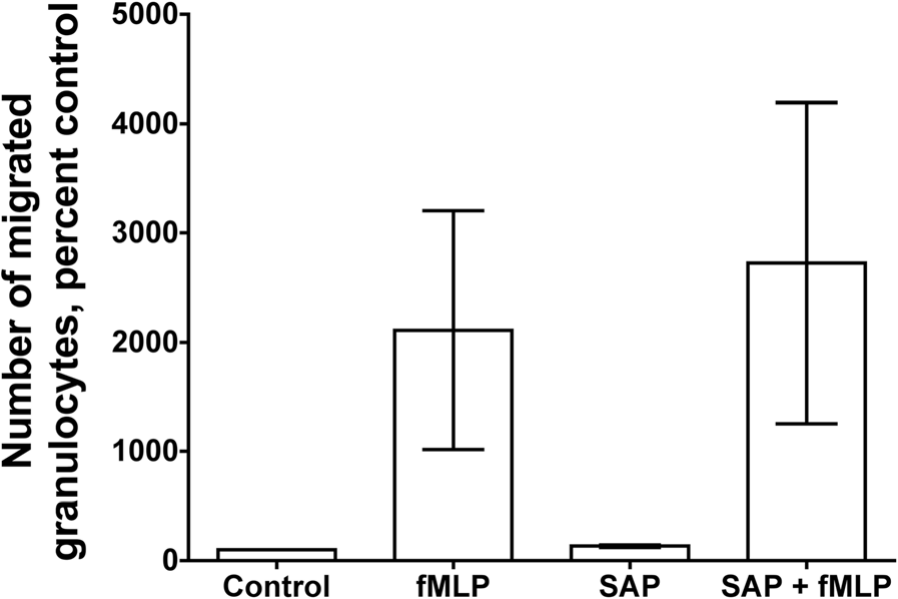
**Serum amyloid P (SAP) has no significant effect on the migration of granulocytes.** Granulocytes were placed on a porous membrane that touched a chamber containing buffer, *N*-formyl-methionine-leucine-phenylalanine (fMLP), SAP, or fMLP and SAP. Migration was carried out for 2 h at 37°C. A total of 25 μl of granulocytes from the bottom chamber was counted with a flow cytometer. The results are mean ± SEM of migrated granulocytes (n = 4 separate experiments).

The formyl peptide fMLP induces the migration of granulocytes [74]. To determine the role of SAP in granulocyte migration, we carried out migration assays using a Boyden chamber. Granulocytes were placed on a porous membrane and the bottom of the membrane touched a solution containing buffer or fMLP in the presence or absence of SAP. fMLP significantly increased the number of granulocytes that migrated across the porous membrane, while SAP had no effect on the migration of granulocytes or the migration of granulocytes caused by fMLP (Figure 9).

### SAP has no effect on the apoptosis of granulocytes

**Figure 10:**
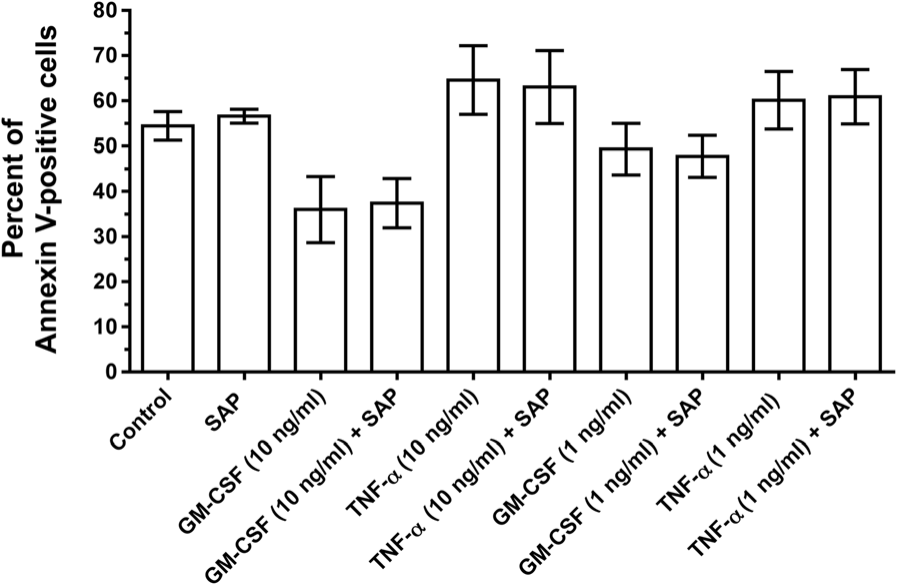
**Serum amyloid P (SAP) has no effect on granulocyte apoptosis.** Granulocytes were treated with granulocyte macrophage colony-stimulating factor (GM-CSF) (10 ng/ml or 1 ng/ml) or tumor necrosis factor (TNF)α (10 ng/ml or 1 ng/ml) in the presence or absence of SAP (60 μg/ml) for 22 h at 37°C. The cells were then stained with annexin V and analyzed by flow cytometry. The results are mean ± SEM of percentage positive cells (n = 3 separate experiments).

Phosphatidylserine gets exposed from the inner surface of the plasma membrane during apoptosis where there is loss of phospholipid asymmetry [75]. Annexin V is a protein that binds to phosphatidylserine, and we can use a fluorescent dye conjugated to annexin V to identify apoptotic cells [76]. Granulocytes were treated with TNFα or GM-CSF in the presence or absence of SAP, and the cells were stained with Alexafluor 488-conjugated annexin V. There was no significant effect on the percentage of annexin V-stained cells in all of the conditions except for the cells treated with 10 ng/ml GM-CSF(Figure 10). GM-CSF-treated cells had a decreased percentage of annexin V-positive cells compared to other conditions, which matches previous observations that GM-CSF delays apoptosis of granulocytes such as neutrophils [77]. SAP had no significant effect on the percentage of annexin V-positive cells under all tested conditions, suggesting that SAP has no obvious effect on granulocyte apoptosis.

### SAP specifically inhibits the accumulation of Ly6G-positive cells in lungs of mice treated with bleomycin

**Figure 11:**
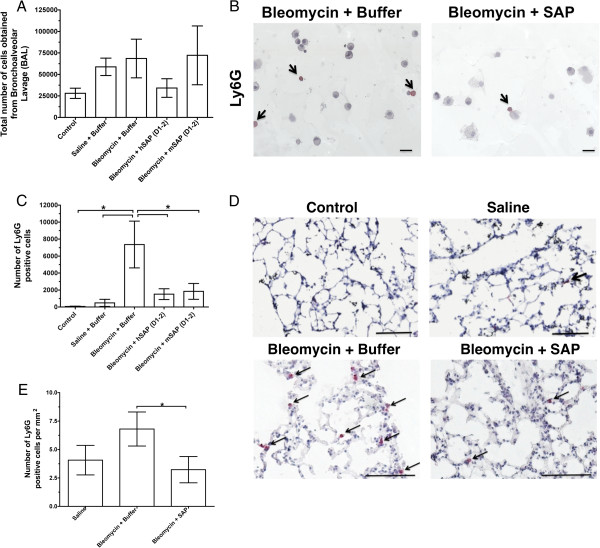
**Serum amyloid P (SAP) decreases the accumulation of Ly6G**-**positive cells in 0.2 U/kg bleomycin-treated mouse lungs.** Mice were treated with 0.2 U/kg bleomycin using an oropharyngeal technique on day 0. Mice were then injected with either 50 μg SAP or an equal volume of buffer on days 1 and 2. After the mice were killed on day 3, cells were collected by bronchoalveolar lavage (BAL). (**A**) The total number of cells collected by BAL from mice with the indicated treatments. (**B**) Cells collected by BAL were stained with anti-mouse Ly6G to detect granulocytes. Arrows indicate Ly6G-positive cells. Bars are 20 μm. (**C**) The total number of Ly6G-positive cells in the above experimental groups. Values are mean ± SEM (n = 4 for control and mice treated with bleomycin and buffer, n = 5 for mice treated with saline and buffer or mice treated with bleomycin and mouse SAP, and n = 6 for mice treated with bleomycin and human SAP). Using the non-parametric Mann–Whitney two-tailed t test, there was an increased number of Ly6G-positive cells in the BAL from bleomycin and buffer when compared to control, mice treated with saline and buffer, or mice treated with bleomycin and human SAP; *P <0.05. Using the non-parametric Mann–Whitney two-tailed t test, there was no significant difference between mice treated with bleomycin and buffer or mice treated with bleomycin and mouse SAP. However, using the non-parametric Mann–Whitney one-tailed t test, there was a statistically significant increase in the number of Ly6G-positive cells in the BAL from mice treated with bleomycin and buffer when compared to the number of Ly6G-positive cells in the BAL from mice treated with bleomycin and mouse SAP. (**D**) After obtaining cells by BAL, lung sections were stained with anti-mouse Ly6G to detect granulocytes. Arrows indicate Ly6G-positive cells. Bars are 100 μm. (**E**) Counts of granulocytes in sections. Values are mean ± SEM, n = 3. *P <0.05 (t test).

Since SAP inhibits granulocyte adhesion, we examined the effect of SAP on bleomycin-induced granulocyte accumulation in the lungs of mice. Mice were treated with or without 0.2 U/kg bleomycin on day 0 and were then given intraperitoneal injections of SAP on days 1 and 2. On day 3, the mice were killed and cells from the lungs were collected by BAL. There was no statistically significant difference in the number of cells collected from BAL of control, saline, 0.2 U/kg bleomycin and buffer, 0.2 U/kg bleomycin and human SAP, or 0.2 U/kg bleomycin and mouse SAP (Figure 11A). Cells collected from the BAL of mice were then stained with anti-mouse Ly6G (this stains neutrophils and some eosinophils) [78,79]. There was an increased number of Ly6G-positive cells in the BAL from mice treated with 0.2 U/kg bleomycin and buffer compared to the number of Ly6G-positive cells in the BAL from control (untreated mice) or saline-treated mice (Figure 11C). However, there was a decreased number of Ly6G-positive cells in the BAL from mice treated with 0.2 U/kg bleomycin and human SAP or 0.2 U/kg bleomycin and mouse SAP when compared to the BAL from mice treated with 0.2 U/kg bleomycin and buffer (Figure 11B,C). There was no significant difference between the number of Ly6G-positive cells in the BAL from mice treated with 0.2 U/kg bleomycin and human SAP or 0.2 U/kg bleomycin and mouse SAP when compared to control (untreated mice) or saline-treated mice. We stained lung sections with Ly6G after BAL to detect granulocytes remaining in the lungs after BAL. There was an increased number of Ly6G-positive cells in the lung sections after BAL in mice treated with 0.2 U/kg bleomycin and buffer compared to the number of Ly6G-positive cells in the lung sections from control (untreated mice), saline-treated mice, or mice treated with 0.2 U/kg bleomycin and human SAP (Figure 11D,E).

**Figure 12:**
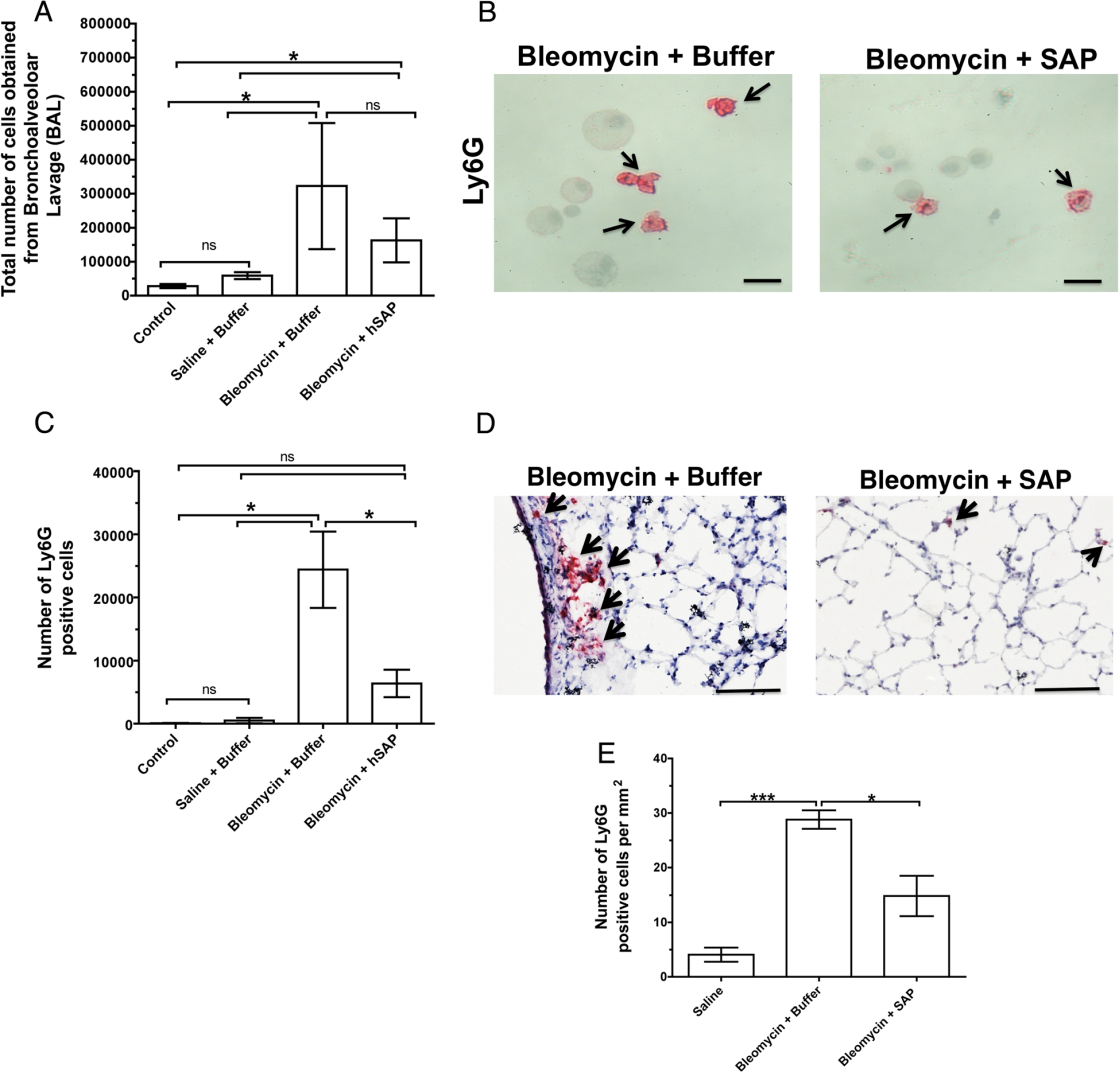
**Serum amyloid P (SAP) decreases the accumulation of Ly6G-positive cells in 3 U/kg bleomycin-treated mouse lungs.** Mice were treated with 3 U/kg bleomycin using an oropharyngeal technique on day 0. Mice were then injected with either 50 μg SAP or an equal volume of buffer on days 1 and 2. After the mice were killed on day 3, cells were collected by bronchoalveolar lavage (BAL). (**A**) The total number of cells collected by BAL from mice with the indicated treatments. (**B**) Cells collected by BAL were stained with anti-mouse Ly6G to detect granulocytes. Arrows indicate Ly6G-positive cells. Bars are 20 μm. (**C**) The total number of Ly6G-positive cells in the above experimental groups. Values are mean ± SEM (n = 4 for control, mice treated with bleomycin and buffer, or mice treated with bleomycin and human SAP, and n = 5 for mice treated with saline and buffer); *P <0.05 (significant difference) as determined by non-parametric Mann–Whitney two-tailed t tests. (**D**) After obtaining cells from BAL, lung sections were stained with anti-mouse Ly6G to detect granulocytes. Arrows indicate Ly6G-positive cells. Bars are 100 μm. (E) Counts of granulocytes in sections. Values are mean ± SEM, n = 3. *P <0.05, ***P <0.001 (t test).

We also similarly treated mice with 3 U/kg bleomycin. Previous results from our laboratory have shown that human SAP is more potent at inhibiting the differentiation of monocytes to fibrocytes than mouse SAP [51], so we decided to only use human SAP. There was no statistically significant difference between the number of cells collected from the BAL of control or saline-treated mice (Figure 12A). However, there were an increased number of cells collected in the BAL from mice treated with 3 U/kg bleomycin and buffer or 3 U/kg bleomycin and human SAP when compared to control or saline-treated mice (Figure 12A). There was also an increased number of Ly6G-positive cells in the BAL from mice treated with 3 U/kg bleomycin and buffer when compared to the number of Ly6G-positive cells in the BAL from control or saline-treated mice (Figure 12B,C). There was a decreased number of Ly6G-positive cells in the BAL from mice treated with 3 U/kg bleomycin and human SAP when compared to the number of Ly6G-positive cells in the BAL from mice treated with 3 U/kg bleomycin and buffer (Figure 12B,C). However, there was no statistically significant difference in Ly6G-positive cells in the BAL from mice treated with 3 U/kg bleomycin and human SAP when compared to the Ly6G-positive cells in the BAL from control or saline-treated mice (Figure 12C). When lung sections were stained with Ly6G after BAL to detect granulocytes remaining in the lungs after BAL, there was an increased number of Ly6G-positive cells in the lung sections of mice treated with 3 U/kg bleomycin and buffer compared to the number of Ly6G-positive cells in the lung sections from control (untreated mice), saline-treated mice, or mice treated with 3 U/kg bleomycin and human SAP (Figure 12D,E).

**Figure 13:**
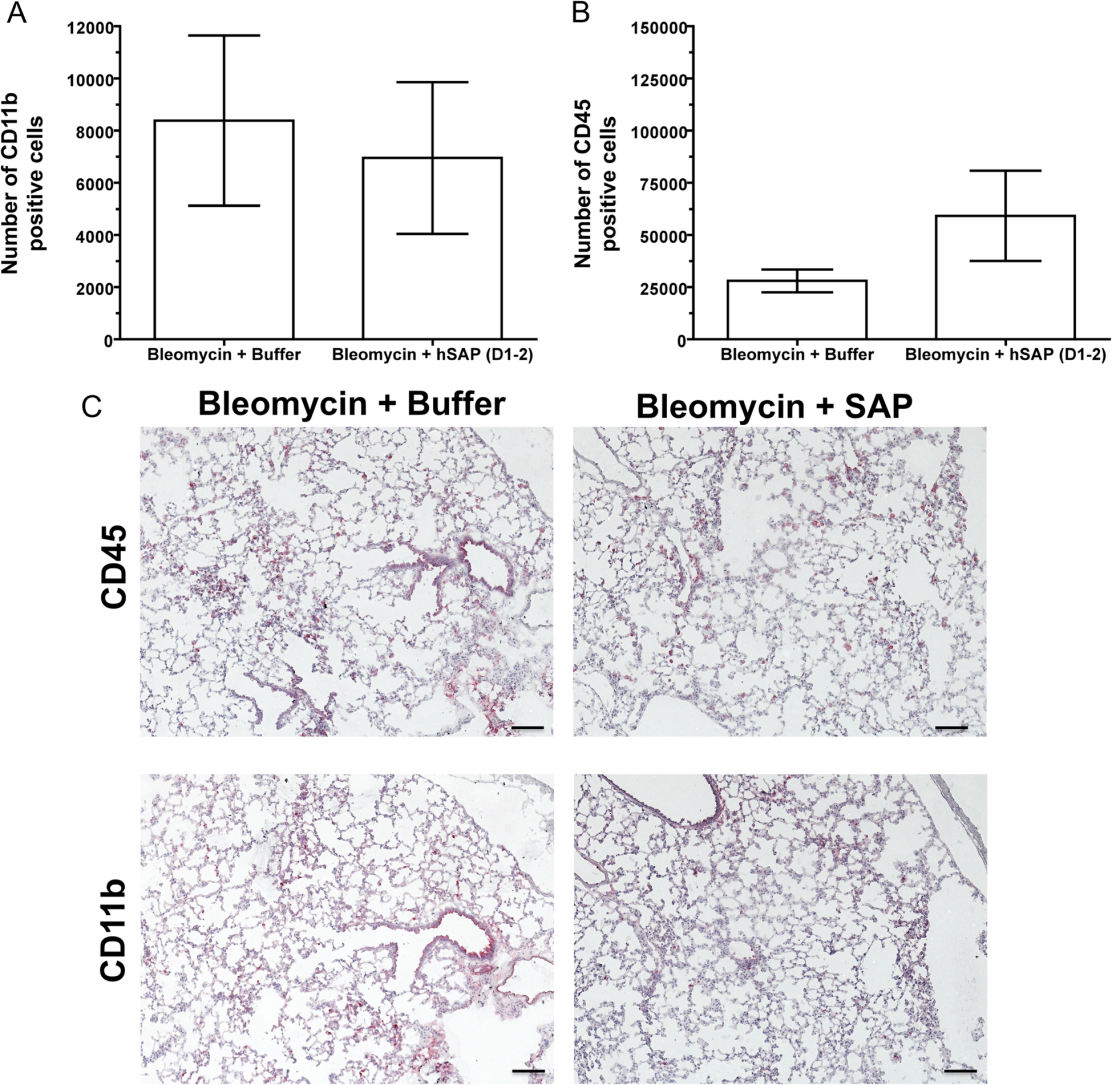
**Serum amyloid P (SAP) has no effect on the accumulation of CD11b**-**positive cells or CD45**-**positive cells in 0**.**2 U**/**kg bleomycin**-**treated mouse lungs.** Cells from bronchoalveolar lavage (BAL) were further analyzed for the presence of CD11b-positive or CD45-positive cells. (**A**) The total number of CD11b-positive cells in the cells collected from the BAL of mice treated with bleomycin and buffer or mice treated with bleomycin and human SAP. (**B**) The total number of CD45-positive cells in the cells collected from the BAL of mice treated with bleomycin and buffer or mice treated with bleomycin and human SAP. (**C**) After obtaining cells from BAL, lung sections were stained with anti-mouse CD11b or CD45 to detect macrophages and leukocytes remaining in the lungs. Arrows indicate CD11b-positive or CD45-positive cells. Bars are 100 μm.

For both 0.2 and 3 U/kg bleomycin followed by buffer injections, there were more granulocytes observed in the lung sections compared to the lung sections from the bleomycin followed by human SAP treatment group (Figures 11D,E and 12D,E). This indicates that if we had been able to obtain all of the pulmonary granulocytes, the difference between bleomycin/buffer injections and bleomycin/SAP injections would have been even greater than the differences shown in Figures 11C and 12C. We further analyzed the lung sections for macrophages and leukocytes using anti-CD11b (in mice, Cd11b is a macrophage marker) and anti-CD45 antibodies (Figure 13). When lung sections were stained for CD11b or CD45, there was no difference between CD11b-positive cells or CD45-positive cells in the lung sections from mice treated with 0.2 U/kg bleomycin and buffer or mice treated with 0.2 U/kg bleomycin and human SAP (Figure 13). Our results therefore suggest that at 3 days, SAP has no effect on the bleomycin-induced accumulation of macrophages or other leukocytes in the lungs, but SAP reduces the bleomycin-induced accumulation of granulocytes in the lungs. However, SAP injections do reduce bleomycin-induced leukocyte infiltration by day 14 or day 21 [52]. It is possible that at an early injury stage, SAP only affects the infiltration of granulocytes, but at later injury stage, SAP can inhibit the overall leukocyte infiltration induced by bleomycin.

## Discussion

We found that SAP inhibits cell debris-induced granulocyte spreading and TNFα-induced granulocyte adhesion on different extracellular matrices. However, SAP has no effect on the surface levels of granulocyte adhesion molecules such as CD11b, CD62L, CD18, or CD44 that are affected by granulocyte activating factors such as TNFα, GM-CSF, or fMLP. SAP also has no effect on the production of hydrogen peroxide induced by granulocyte activating factors such as PMA, PDBu, fMLP, or TNFα. In addition, SAP did not have a significant effect on fMLP-induced granulocyte migration. Nevertheless, intraperitoneal injections of SAP significantly reduced the number of granulocytes that accumulate in the lungs of mice treated with bleomycin.

A previous report found that SAP acts as a granulocyte chemoattractant, increases granulocyte adhesion, and increases the percentage of granulocytes expressing CD11b, CD18, and α5β1 [58]. However, a different group found that SAP inhibits the binding of human granulocyte to TNFα-stimulated human umbilical vein endothelial cells [59]. In this report, we find that SAP does not act as a granulocyte chemoattractant, tends to decrease granulocyte adhesion, and does not affect the percentage of granulocyte expressing CD11b and CD18. Given that a variety of factors can activate granulocytes, induce granulocyte chemotaxis and increase granulocyte adhesion, we hypothesize that the SAP used in the first report may have been contaminated with a small amount of some material that activated the granulocytes.

Other pentraxin family proteins also inhibit granulocytes such as neutrophil accumulation in animal models of acute lung injury (ALI) or acute respiratory distress syndrome (ARDS) [37]. CRP and PTX3 also decrease the number of neutrophils that accumulate in injured lungs [32,37]. CRP inhibits neutrophil adhesion and chemotaxis [37]. Administering CRP intravenously 10 minutes before the intratracheal instillation of the neutrophil chemotactic agent C5a reduces neutrophil accumulation in lungs [35]. CRP inhibits L-selectin-mediated neutrophil adhesion on TNFα activated endothelial cells by inducing L-selectin shedding from neutrophils. CRP peptide 201–206 mediates the antiadhesive action through CD32 [33]. Both native CRP and CRP peptide 201–206 prevent neutrophil chemotaxis towards fMLP by inhibiting fMLP-induced p38 mitogen-activated protein (MAP) kinase activity [36]. Similarly, pretreating mice intravenously with PTX3 reduces the number of neutrophils in acid-induced acute lung injury in mice [32]. PTX3 deficiency also increases the number of neutrophils in the lungs of mice treated with lipopolysaccharide (LPS) [34]. PTX3 blocks the interaction of P-selectin glycoprotein ligand-1 (PSGL-1) on neutrophils from interacting with P-selectin on the activated endothelial cells and causes neutrophil detachment rather than arrest to prevent neutrophil migration [32]. An intriguing possibility is that the three pentraxins (SAP, CRP, and PTX3) may use a common mechanism to inhibit granulocytes such as neutrophil adhesion.

SAP prevents the accumulation of granulocytes in the lungs of bleomycin-injured mice. Since SAP inhibits granulocytes adhesion, it is probable that SAP reduces the accumulation of granulocytes by dampening the interaction of granulocytes with extracellular matrices. We still do not know the granulocyte adhesion receptors that are affected by SAP. One possibility is that SAP affects β1 integrins such as α2β1, α5β1, α6β1, or α9β1 that are found on granulocytes and can recognize different extracellular matrices [80-83].

## Conclusions

We found that SAP, a constitutive component of blood, is a granulocyte adhesion inhibitor. Furthermore, we found that injections of SAP decrease granulocyte levels in the lungs in a murine model of ARDS. We hypothesize that SAP allows granulocytes to sense whether they are in the blood or in a tissue, and that increasing serum SAP levels, for instance by injection, may be a possible therapeutic for neutrophil-associated diseases such as ARDS.

## Abbreviations

ARDS: Acute respiratory distress syndrome; BAL: Bronchoalveolar lavage; BSA: Bovine serum albumin; CRP: C-reactive protein; fMLP: *N*-formyl-methionine-leucine-phenylalanine; GM-CSF: Granulocyte macrophage colony stimulating factor; ICAM-1: Intracellular adhesion molecule-1; IL: Interleukin; KRPG: Krebs-Ringer phosphate glucose buffer; LXA4: Lipoxin A4; LXB4: Lipoxin B4; PTX3: Pentraxin-3; PBMCs: Peripheral blood mononuclear cells; PMA: Phorbol 12-myristate 13-acetate; PDBu: Phorbol 12,13-dibutyrate; PSGL-1: P-selectin glycoprotein ligand-1; SAP: Serum amyloid P; SFM: Serum-free media; TNFα: Tumor necrosis factor α

## Competing interests

RHG, DR and ASM filed an invention disclosure to Texas A&M University and Rice University on the use of SAP to inhibit neutrophil adhesion and as a possible therapy for acute respiratory distress syndrome and other neutrophil-driven diseases. The two universities have filed a provisional patent application on this disclosure. In addition, RHG is a coinventor on patents for the use of SAP as a therapeutic for fibrosing diseases, and patents on the use of SAP-depleting materials to enhance wound healing. RHG is a member of the Science Advisory Board of, and has stock options from, Promedior, a start-up company that is developing serum amyloid P as a therapeutic for fibrosing diseases, and receives a share of milestone payments made by Promedior to Rice University.

## Authors’ contributions

ASM conceived and designed experiments, performed experiments, analyzed data and wrote the paper. DR conceived and designed experiments, performed experiments, and analyzed data. DB conceived and designed experiments, performed experiments, and analyzed data. RHG conceived and designed experiments, performed experiments, analyzed data and edited the manuscript. All authors read and approved the final manuscript.
